# NK-Like T Cells and Plasma Cytokines, but Not Anti-Viral Serology, Define Immune Fingerprints of Resilience and Mild Disability in Exceptional Aging

**DOI:** 10.1371/journal.pone.0026558

**Published:** 2011-10-20

**Authors:** Abbe N. Vallejo, David L. Hamel, Robert G. Mueller, Diane G. Ives, Joshua J. Michel, Robert M. Boudreau, Anne B. Newman

**Affiliations:** 1 Department of Pediatrics, University of Pittsburgh School of Medicine, Children's Hospital of Pittsburgh, University of Pittsburgh Medical Center, Pittsburgh, Pennsylvania, United States of America; 2 Department of Immunology, the University of Pittsburgh Cancer Institute, and the McGowan Institute for Regenerative Medicine, University of Pittsburgh, Pittsburgh, Pennsylvania, United States of America; 3 Medical Student Training in Aging Research Program, Division of Geriatric Medicine, University of Pittsburgh School of Medicine, Pittsburgh, Pennsylvania, United States of America; 4 Center for Aging and Population Health, Department of Epidemiology, University of Pittsburgh Graduate School of Public Health, Pittsburgh, Pennsylvania, United States of America; New York University, United States of America

## Abstract

Exceptional aging has been defined as maintenance of physical and cognitive function beyond the median lifespan despite a history of diseases and/or concurrent subclinical conditions. Since immunity is vital to individual fitness, we examined immunologic fingerprint(s) of highly functional elders. Therefore, survivors of the Cardiovascular Health Study in Pittsburgh, Pennsylvania, USA were recruited (n = 140; mean age = 86 years) and underwent performance testing. Blood samples were collected and examined blindly for humoral factors and T cell phenotypes. Based on results of physical and cognitive performance testing, elders were classified as “impaired” or “unimpaired”, accuracy of group assignment was verified by discriminant function analysis. The two groups showed distinct immune profiles as determined by factor analysis. The dominant immune signature of impaired elders consisted of interferon (IFN)-γ, interleukin (IL)-6, tumor necrosis factor-α, and T cells expressing inhibitory natural killer-related receptors (NKR) CD158a, CD158e, and NKG2A. In contrast, the dominant signature of unimpaired elders consisted of IL-5, IL-12p70, and IL-13 with co-expression of IFN-γ, IL-4, and IL-17, and T cells expressing stimulatory NKRs CD56, CD16, and NKG2D. In logistic regression models, unimpaired phenotype was predicted independently by IL-5 and by CD4^+^CD28^null^CD56^+^CD57^+^ T cells. All elders had high antibody titers to common viruses including cytomegalovirus. In cellular bioassays, T cell receptor (TCR)-independent ligation of either CD56 or NKG2D elicited activation of T cells. Collectively, these data demonstrate the importance of immunological parameters in distinguishing between health phenotypes of older adults. NKR^+^ T cells and cytokine upregulation indicate a unique physiologic environment in old age. Correlation of particular NKR^+^ T cell subsets and IL-5 with unimpaired performance, and NKR-driven TCR-independent activation of T cells suggest novel immunopathway(s) that could be exploited to improve immunity in old age.

## Introduction

Older adults aged ≥65 years have very heterogeneous health characteristics. They range from the very frail to those with exceptional physical and cognitive function despite long history of disease and concurrent subclinical conditions [1], [2]. Immunologically, they range from the immunocompromised to those who mount vigorous responses to vaccination [3], [4], [5]. Since immunity is a determinant of individual fitness, it is reasonable that favorable health in late life could be mediated by mechanism(s) of immune homeostasis distinct from that seen at early adulthood to mid-life, akin to documented differences in immune protective mechanisms between neonates and adults [6]. This implies normal age-specific differences in immune physiology consistent with developmental changes that organ-systems normally undergo through the lifespan [7], [8].

Differences in immune responsiveness between older adults and younger persons are associated with age-related changes in the T cell repertoire. Production of new naïve T cells is impaired in older adults due to the involution to the thymus. Exposure of T cells to pathogens through life contributes to the depletion of the naïve T cell pool and to the overall expansion of memory cells with contracted diversity of the repertoire of T cell receptors (TCR) due to the over representation of oligoclonal T cells. Indeed, poorer antigen-specific responses to the vaccine against seasonal influenza in the elderly has been recently linked to the contraction of TCR diversity [9].

There is an emerging biological theme for a secondary level of T cell diversity with advancing age. Several investigators have shown increased expression of a variety of natural killer cell-related receptors (NKR) on T cells of older individuals [10], [11]. T cell clones, verified by identical *TCR-CDR3* DNA sequences, that express different repertoires of NKRs have been isolated from peripheral blood [12]. In addition, NKRs are co-dominantly expressed and are found in various combinations on T cells [13], [14], [15]. Thus, the aged T cell repertoire could remain diverse at the level of NKR expression along T cell clonal lineages, in marked distinction from the repertoire of the young that is diverse at the level of the clonotypic TCR. In recent work, we reported that increased expression of the prototypical NKR, CD56, on T cells with chronologic aging endows functional competence to such aged T cells [16]. All these observations are consistent with the idea that the T cell repertoire undergoes remodeling with advancing age [17]. While there are clear negative immunological changes with usual chronologic aging [18], [19], [20], T cell repertoire remodeling implies that late life survival need not be synonymous with ill-health or immune incompetence. We suggest that the nature and extent of repertoire remodeling influences health outcomes in old age.

To further evaluate this idea of beneficial repertoire remodeling, we examined a subset of community-dwelling elderly persons who are survivors of the Cardiovascular Health Study (CHS), called “CHS All Stars” [21]. Given that CHS All Stars elders have a mean age of 86 years who are surviving ∼10 years beyond the American median lifespan of 77 years [22], we hypothesized that those who maintain high functional performance would have a distinctive immunological profile. We sought to identify novel functionally active T cell subsets and/or humoral factors that distinguish highly functional elders from those with physical or cognitive impairment.

## Materials and Methods

### Protection of human subjects

Human subjects research was conducted in accordance with principles expressed by the Declaration of Helsinki. Studies described in this work were in compliance with protocols approved by the Institutional Review Board of the University of Pittsburgh. All subjects provided written informed consent, and biological specimens and all data collected were anonymized.

### Subject enrollment, health data collection, and performance testing

The CHS is a multi-center longitudinal large-cohort study of 5,888 men and women recruited in 1989–90 and an added minority cohort in 1992–93 [23]. At the Pittsburgh Field Center, there are 258 survivors of the CHS, called “CHS All Stars”, who were visiting for the 18^th^ year of follow up [21]. Of this group, a consecutive sample of 140 participants was consented to contribute additional blood samples for this study. At the time of this examination, all participants were community dwellers, and were aged 78 to 94 years with only 2 individuals who were <82 years old (mean age of 86 years old). Health information were collected using CHS-standardized protocols that included a comprehensive characterization of health and function, medical history and hospitalizations, and self-rating of health [23], [24], [25]. Demographic information such as age, sex, race, height, weight, and body mass index were also collected.

All subjects underwent new performance testing of cognitive and physical ability. Global cognitive function was evaluated by the modified 100-point scale mini-mental examination (3MSE) [26]. Physical function was examined by measurements of grip strength and gait speed [27], [28], and by a self-report of difficulty in performing activities of daily living (ADL), namely; eating, transferring, dressing, toileting, and bathing [29]. Physical impairment was defined as self-report of any difficulty with ADL. Cognitive impairment was defined as having a 3MSE score of less than 80. “Impaired” status was defined as having either physical and/or cognitive impairment, and “unimpaired” as having neither physical nor cognitive impairment [30], [31].

As internal reference controls, we also examined banked specimens obtained from young adults aged 18–40 years that were part of a previous cross sectional study [16]. These younger subjects were screened previously for the absence of any current or historical diagnosis of chronic inflammatory disease, organ transplant, or malignancy, and/or use of immunosuppressive drugs, chemotherapy, or oxygen. They served mainly as sources of peripheral blood mononuclear cells (PBMC) that were used as calibration control to more appropriately define T cell phenotypes in flow cytometry procedures, and also as internal positive control in cellular bioassays. As such, they served solely as internal instruments for optimizing experimental procedures, rather than using them as a group to compare functional performance with the elderly subjects.

### Collection of biological specimens

Blood samples were collected during the morning hours; plasma and cell fractions processed on the same day of collection. Plasma aliquots were stored in −80°C until use. PBMC were isolated by standard isopycnic centrifugation over Ficoll gradient, and cells were stained and analyzed by flow cytometry (see below). Aliquots of PBMC were also cryopreserved for re-analysis of cell phenotypes as needed to ensure reproducibility of results, and as sources of materials for biological assays (see below). As needed, cryofrozen PBMC were thawed on ice and used immediately. Plasma samples were similarly thawed and used at no more than two freeze/thaw cycles.

### Analysis of plasma cytokines and anti-viral antibodies

Classical inflammatory mediators such as interleukin (IL)-1, IL-6, tumor necrosis factor (TNF)-α, and reactant C-reactive protein (CRP) have been associated with age-related maladies including physical/cognitive disability [32]. Here, we conducted a global analysis of humoral factors to examine additional molecules associated with poorer health, as well as with good health. Assays for cytokines were performed using Human Cytokine 17-plex panel kit (BioRad) according to manufacturer specifications. Cytokine concentrations were obtained using the Luminex 100 system (Luminex Corp). CRP was measured independently by quantitative enzyme-linked immunosorbent assay (ELISA) using a commercial kit (R&D Systems).

Seropositivity to cytomegalovirus (CMV) has been reported to be a predictor of ill-health in some Northern European elderly populations [33]. Hence, titers of antibodies to CMV, and three other common viruses, varicella zoster virus (VZV), Epstein-Barr virus (EBV), and influenza common hemagglutinin (FluHA) in plasma samples were measured by quantitative ELISA using diagnostic kits. Anti-CMV titers were determined using CMV immunoglobulin G (IgG) Enzyme Immunoassay kit (MP Biochemicals); a propriety kit-software was used to generate a best fit equation (R^2^≥0.95) from which sample anti-CMV titers were calculated. Anti-VZV and anti-FluHA were measured using VZV IgG and Flu-IA IgG ELISA kits (Diagnostic Automation), respectively; quantitative determination of antibody titers was performed using a lot-specific formula provided by the manufacturer. Anti-EBV early antigen (EA) IgG and IgM, and to EBV Nuclear Antigen (EBNA) IgG were measured using specific kits (Biotest); quantitative determination of antibody titers achieved using a standard curve and a best-fit equation (R^2^≥0.95) as specified by the manufacturer.

### Analysis of T cell populations

For this study, leukocyte population analysis was limited to T cells. Cell phenotypes were examined by multicolor flow cytometry using a previously described procedure [16]. Briefly, an aliquot of PBMC were stained with fluorochrome-conjugated antibodies (BD; Ebioscience; BioLegend; Abcam; Beckman Coulter) to classical T cell markers TCRαβ, CD3, CD4, CD8αβ, and CD28; to the adhesion molecule CD57; to the NKRs CD16, CD56, NKG2D, NKG2A, CD158a, CD158b, and CD158e; to senescence antigens p16, p53, phospho-γH2AX, and pRB, and to B cell and monocyte markers CD19 and CD14, respectively, in order to exclude contamination of T cell populations of interest. Each cytometry experiment included control cells singly stained for each molecule, and fluorochrome-conjugated beads for instrument calibration and for off-line calculation of signal compensation. Raw cytometry data were acquired using the LSRII cytometer (BD).

Cytometry data were analyzed offline using FlowJo software (Tree Star). Signal compensation was applied using the appropriate bead controls. Debris and cell doublet events were electronically filtered out using scatter parameters, and a live lymphocyte gate was determined using standard forward and scatter signals. Frequency of cells expressing specific markers was recorded. For each NKR examined, the density of expression was also measured as mean fluorescence intensity (MFI) or geometric MFI (GMFI). The latter was measured so as to minimize effects of counting cells with extremely high or extremely low receptor density.

### Bioassays of T cell function

T cell responses depend on signals from other cellular components of the immune system. Since chronologic aging is associated with varying degrees of alterations in the numbers, phenotypes, and function of immune cell subsets [11], [18], [19], [20], unfractionated PBMC was used in bioassays in order to minimize false negative results that could arise from the imbalance of cellular components when particular cell subsets are removed in the assay. Considering intrinsic variations of T cell subsets among subjects [16], this experimental strategy rendered each subject as a control to itself, and also ensured evaluable number of cells. Another advantage of this strategy is the utilization of constituent, unmanipulated, autologous monocytes as surrogate antigen presentors that bound antibodies or Ig-Fc fusion proteins used for T cell stimulation, and provided necessary costimulatory factors.

PBMC were suspended in RPMI 1640 culture medium and plated out at 1−10×10^5^ cells/well in 96-well tissue culture plates with immobilized stimulatory antibodies or IgG controls or recombinant ligands according to established protocols [34]. Based on empirical experiments, optimal stimulatory conditions were 1 µg/ml of anti-TCRαβ (1P26, Biolegend) or anti-CD3 (OKT3, Orthoclone) or anti-CD56 (NCAM, Abcam or C218, Beckman Coulter), or anti-NKG2D (1D11, Ebioscience), or 10 ng/ml of recombinant NKG2D ligands (R&D Systems), namely, MIC-A-Fc, ULPB1-Fc, ULPB2-Fc, and ULPB3-Fc [35]. After 24 hours in tissue culture, cells were harvested. CD4^+^ and CD8^+^ T cells were examined for the expression of activation antigens CD25 and CD69, the granule exocytosis markers CD107a and CD107b, the cytolytic granules perforin and granzyme, and the cytokines interferon (IFN)-γ and IL-4 by multicolor flow cytometry (as described above). For the detection of the latter four cytoplasmic molecules, cultures were incubated with a monensin-brefeldin A cocktail (Golgi Stop/Golgi Plug, BD) for 4–6 hours prior to harvest in order to prevent vesicle exocytosis. For this study, we combined antibodies to perforin and to granzyme tagged with the same fluorochrome to detect expression of both cytolytic molecules.

MFI of each of molecules were used to determine the stimulation index (SI) of cellular response that was calculated by the formula: SI = (MFI of test sample - background MFI)/MFI of control. Because the numbers of T cells expressing CD56 or NKG2D vary between subjects, SI was normalized to the proportion of CD56^+^ or NKG2D^+^ T cells for each subject sample.

### Statistical Analyses

All analyses were performed using PASW Version 18 software (SPSS Inc). Preliminary inter-variable relationships were examined by Spearman's rank correlation. Preliminary assessment of differences between the two pre-defined categorical groups of subjects, i.e. “impaired” and “unimpaired”, was examined by descriptive statistics including mean, median, mode, range, and coefficient of variation. Boxplots were used to visualize any between-group differences. Chi-square test or Mann-Whitney U-test was used as appropriate to compare group means. For T cell bioassays, Kruskal-Wallis analysis of variance was used to compare means of independent variables.

Discriminant function analysis using immune profiles was performed to evaluate overall confidence for the assignment of subjects to the two subject-categories. Both simple linear and stepwise models of subject-group discrimination were evaluated. To ultimately determine if clusters of immunologic parameters specifically differentiated the unimpaired group from the impaired group, factor analysis by Varimax rotation with Kaiser normalization was performed. Whereas a significant cluster in factor analysis is generally defined by an Eigen value >1.0, we used a more stringent Eigen value ≥5.0 to identify principal components that were more highly significant. Principal components comprising >75% of cumulative Eigen value (CEV) were considered as main factors that distinguished between the impaired and unimpaired groups. Those immunologic parameters with rotated component factor loading coefficient of ≥0.70 in absolute value (i.e. those whose correlation with the factor that were at least 0.7) were identified as those characterizing a factor or cluster. Subsequently, logistic regression (backward and forward stepwise models) was performed to generate odds ratios of predictors for membership in the unimpaired categorical group. Significant odds ratios were defined with a 95% confidence interval. For all statistical measures, P-values <0.05 were considered significant.

## Results

### CHS All-Star elders exhibit classic features of T cell aging

CHS All Stars elders have a mean age of 86 years (see below), surviving ∼10 years beyond the American median lifespan of ∼77 years [22]. Hence, we first examined all subjects for general features of immune aging in the T cell compartment, namely, the irreversible loss of CD28, and the gain of CD57 expression [36], [37]. Since it is not yet known whether or not CD28 loss and CD57 gain on T cells is controlled by the same or by independent pathways, we separately analyzed T cell subsets based on expression of these two antigens.

**Figure 1:**
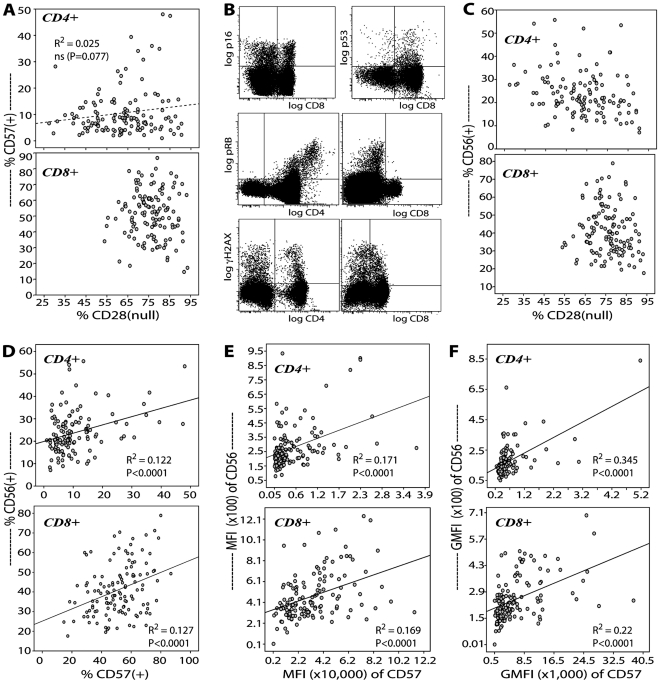
CHS All Stars elders display classic features of aging in the T cell compartment. By multicolor flow cytometry, CD3^+^ TCRαβ T cells in PBMC were examined for senescence-associated loss of expression of the costimulatory receptor CD28, and gains of expression of the adhesion molecule CD57 and the prototypic NKR CD56, as well as the expression of senescence antigens p16, p53, pRB, and γH2AX. (**A**) Scatter plot of the frequencies of CD28^null^ and CD57^+^ cells in the CD4 and CD8 T cell compartments. (**B**) Representative profiles of p16, p53, pRB, and γH2AX expression in CD4 and CD8 T cells. (**C**) Scatter plot of the frequencies of CD28^null^ and CD56^+^ T cells. (**D**, **E**, **F**) Scatter plot of the levels of expression of CD56 and CD57 measured as cell frequencies (D), as MFI (E), and as GMFI (F) in the CD4 and CD8 T cell compartments. Solid and dashed lines represent significant and non-significant regression lines, respectively, with the indicated R^2^ and p-values.

***Figure 1A*** shows that CHS All Stars elders carried large populations of CD28^null^ and CD57^+^ T cells. These unusual lymphocytes occupied up to 94% and 49% of the total CD8 and CD4 compartments, respectively. Whereas CD8 T cells showed clustering of frequencies of CD28^null^ and CD57^+^ cells, there appeared a linear, but not significant, correlation between these two subsets among CD4 T cells. Additional biological indicators of T cell aging are shown in ***Figure 1B*** depicting high number of CD4 and CD8 T cells that expressed the cell senescence antigens p16 and p53 consistent with our previous report [16]. Here, we report that T cells also expressed two other senescence antigens, pRB and phospho-γH2AX. CD4 and CD8 T cells that independently expressed these four cell senescence markers comprised up to 40% of either compartment. There were varying levels of co-expression of these senescence proteins, but it was not apparent whether or not there was dominance of one protein over another.

Aging of human T cells is also accompanied by the increased expression of the prototypic NKR, CD56 [16]. ***Figure 1C*** shows both CD4 and CD8 compartments of CHS All Stars elders contained high frequency of CD56^+^ T cells. Prevalence of CD56^+^ T cells was concordant with higher proportions of CD28^null^ T cells in the CD8 compartment compared to the CD4 compartment. Although it is not yet known whether or not the gains of expression of CD56 and CD57 on T cells with chronologic aging are co-regulated, ***Figure 1D*** shows direct correlation between the frequencies of CD56^+^ and CD57^+^ T cells in both CD4 and CD8 compartments. The quantitative levels of expression of CD56 and CD57, measured either as MFI or GMFI as depicted in ***Figures 1E***–***1F***, respectively, were also directly proportional.

### Characteristics of impaired and unimpaired CHS All Stars elders

***Table 1*** summarizes the characteristics of the two categories of CHS All Stars elders. Consistent with previous clinical assessments of the larger CHS cohort [38], [39], [40], All Stars elders had a wide variety of health characteristics. They had historical and continuous report of co-morbid conditions. They also had varying immunization compliance, and self-rating of health. Both groups had a mean age of 86 years. Blacks, Whites, and both genders were well represented. Both groups had very similar medical history. There were unexpected higher reports of incident cardiovascular disease, diabetes, and arthritis in the unimpaired group, but only incident arthritis was statistically significant compared to the impaired group. Nevertheless, the unimpaired group had significantly higher number who reported with good to excellent health, and a corresponding significantly lower number who reported poor to fair health. Notably, the unimpaired group exhibited significantly better physical performance with higher gait speed and grip strength measurements.

**Table 1:** Demographic and health characteristics of functional categories of CHS All Stars elders.

	Impaired^a^	Unimpaired^a^	P^b^
Total number	58	82	
Sex			
Male, n	17	30	
Female, n	41	52	
Race			
Black, n	18	31	
White, n	40	51	
Mean age (SD)	86.2 (3.3)	85.6 (4.0)	
Mean height, cm (SD)	158 (11)	161 (9)	
Mean weight, lbs (SD)	151 (37)	153 (29)	
Mean BMI^c^ (SD)	27.2 (6.6)	27.8 (4.0)	
Ever smoke, n (%^f^)	36 (62.1)	50 (61.0)	
Medical history, n (%^f^)			
Any cancer	19 (32.8)	24 (29.3)	
Any CVD^c^	38 (65.5)	56 (68.3)	
Type II diabetes	7 (12.1)	15 (18.3)	
COPD^c^	12 (21.7)	14 (17.1)	
Arthritis^d^	26 (44.8)	41 (50.0)	<0.05
Recent vaccination, n (%^f^)			
Influenza vaccine	45 (77.6)	68 (82.9)	<0.05
Pneumococcal vaccine	34 (58.6)	54 (50.0)	<0.05
Self report of health, n (%^f^)			
Poor or Fair	24 (41.3)	23 (28.3)	<0.05
Good to Excellent	34 (58.6)	59 (72.0)	<0.05
Mean gait speed, m/sec (SD)	0.64 (0.1)	0.94 (0.1)	<0.05
Mean grip strength, kg force (SD)	18.1 (0.3)	20.9 (0.4)	

a “Impaired” was defined by 3MSE <80 and/or any reported ADL difficulty. “Unimpaired” was defined by 3MSE score ≥80 and absence of any ADL difficulty.

b P-values determined by Mann-Whitney U-test.

c BMI, body mass index; COPD, chronic obstructive pulmonary disease; CVD, cardiovascular disease inclusive of hypertention, angina, incident myocardial infarction, congestive heart failure, transient ischemic attack, and stroke.

d Arthritis inclusive of rheumatoid and osteoarthritis.

f Percent of the subjects within the group.

### Value of immunological parameters in assigning CHS All Stars elders to impaired and unimpaired performance categories

Data in ***Figure 1*** show that CHS All Stars elders displayed classic predictable features on immune aging. Therefore, we inspected whether there were particular immunological parameters (treated as independent variables) that were associated with the two performance categories of elders. Supplemental data in *Tables S1, S2, S3, S4* show the collated measurements of 12 cytokines, 5 chemokines, 1 acute phase reactant, 6 anti-viral antibody titers, and 88 T cell parameters. For T cells, specific subsets were determined based on the expression of CD28, CD57 and seven NKRs.

The collated humoral data (*Table S1*) showed the impaired group had significantly higher mean values of granulocyte colony stimulating factor, IL-6, IL-12p70, and IFN-γ. The impaired group also had higher mean and median values of IL-7, IL-10, TNF-α, CRP, and macrophage inhibitory protein-1β (MIP-1β), whereas the unimpaired group had higher mean/median values of IL-5; but these latter differences did not reach statistical significance. Regardless of statistical significance, the overall cytokine environment was characterized by the co-expression of Th1 (IFN-γ, IL-12p70), Th2 (IL-4, IL-5, IL-13), and Th17 (IL-17) cytokines. In addition, members of both groups were uniformly seropositive for exposure to CMV, EBV, VZV, and Flu HA.

Overall, CHS All Stars elders showed a normal 3∶1 ratio for classical CD4 and CD8 T cells. They constituted ∼62% of CD3^+^ TCRαβ^+^ lymphocytes. The remaining ∼38% T cells lacked expression of both CD4 and CD8, herein referred to as double-negative or DN T cells (*Table S2*). CD4∶CD8 ratio, and the proportions of CD4, CD8, and DN T cells were not significantly different between unimpaired and impaired elders.

The two groups showed certain pairwise differences in some T cell subsets based on the expression levels of CD28, CD16, CD56, CD57, and NKG2A. The unimpaired group had significantly higher mean frequency of CD4 and CD8 T cells that were CD28^null^, CD57^+^, or CD28^null^CD56^+^CD57^+^. In contrast, the impaired group showed significantly higher mean frequency of CD8 and DN T cells that were NKG2A^+^ (*Table S2*). In addition, the unimpaired group had higher mean/median frequency of NKG2D^+^ CD4 T cells, and the impaired group had higher mean/median frequency of CD158a^+^ and CD158e^+^ CD8 T cells; but these differences were not statistically significant.

The observed differences in T cell frequencies between the two groups were generally concordant with the density of antigen expression (*Table S3* and *Table S4*). The unimpaired group showed significantly higher mean MFI or GMFI values for CD16 and CD57 on CD4 T cells and for CD56 on DN T cells; whereas the impaired group had significantly higher mean GMFI value for NKG2A on CD8 T cells. There were also higher mean/median MFI values for CD158a and CD158e on CD4 and CD8 T cells, and higher GFMI value for CD158a on DN T cells in the impaired group. Similarly, there were higher mean/median MFI values for NKG2D on CD4, CD8, and DN T cells in the unimpaired group. These latter observations however were not statistically significant.

**Figure 2:**
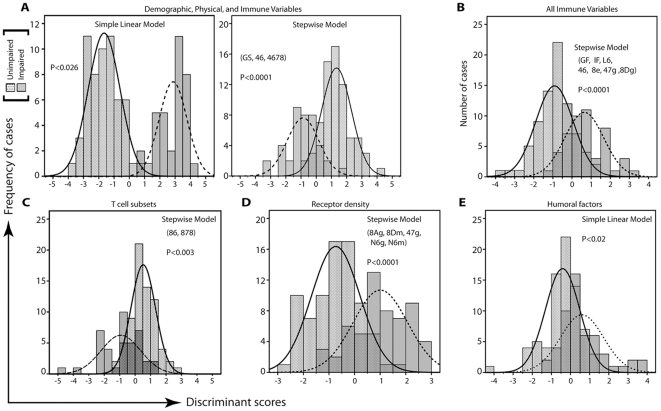
Group assignment of CHS All Stars elders based on 3MSE/ADL scores is discriminated by physical and immune parameters. Discriminant function analysis was performed on collated physical, demographic, and immunological data (see *Table 1* and *Tables S1, S2, S3, S4*). Using all variables, both simple and stepwise models showed significant separation between impaired and unimpaired groups around the centroid, indicated as the zero-mark on the x-axis (**A**). Using only immune variables (**B**), or T cell subset frequency (**C**), or receptor density on T cells (**D**) stepwise models showed significant separation between the two groups despite some overlap. When humoral factors were used only in the analysis, only a simple linear model could be constructed (**E**). Significant contributing variables to models shown in *B, C, D* were: gait speed (GS); the T cell subsets CD4^+^CD56^+^ (46), CD4^+^CD28^null^CD56^+^CD57^+^ (4678), CD8^+^CD158e^+^ (8e), CD8^+^CD56^+^ (86), and CD8^+^CD28^null^CD57^+^ (878); GMFI of CD57 on CD4 T cells (47 g); the GMFI of NKG2D (8 Dg) and NKG2A (8 Ag) on CD8 T cells; the GMFI (N6 g) and MFI (N6 m) of CD56 on DN T cells; the MFI of NKG2D on CD8 T cells (8 Dm); and the humoral factors GMCSF (GF), IFN-γ (IF), IL-6 (L6), and IL-12p70 (L12). The indicated p-values were based on χ^2^ test of Wilk's lambda discriminant statistic.

Discriminant function analysis was performed to determine confidence of subject assignment to either the impaired or the unimpaired group. ***Figure 2A*** shows significant separation between the two groups, with 97.8% of subjects correctly classified (p<0.026) using each physical, demographic, and immunologic data point as independent variables in a simple linear model. As expected, some overlap between the groups was observed in a stepwise model since the relative significance of each contributing variable was considered. But despite the overlap, 90.0% of subjects were deemed correctly classified, with gait speed and subsets of CD4^+^CD56^+^ and CD4^+^CD28^null^CD56^+^CD57^+^ T cells as the most significant contributors to the separation of the two groups (p<0.0001). As depicted, the two groups appeared to be normally distributed either to the left or to the right of the centroid (i.e. at x = 0).

Further stepwise discrimination analysis was performed to assess relevance of immunologic variables alone in assigning elders to impaired and unimpaired groups. ***Figure 2B*** shows a stepwise model with 80.0% correct group-assignment based on immunologic variables only. Significant contributors to this model were granulocyte colony stimulating factor, IFN-γ, IL-6, IL-12p70, frequencies of CD4^+^CD56^+^ and CD8^+^CD158e^+^ T cells, CD57-GMFI on CD4 T cells, and NKG2D-GMFI on CD8 T cells (p<0.0001). ***Figure 2C*** shows a stepwise group-separation model based on T cell subset frequency with 78.7% correct group-assignment. Significant contributors to this model were CD8^+^CD56^+^ and CD8^+^CD28^null^CD57^+^ T cells (p<0.003). ***Figure 2D*** shows a stepwise model with 77.0% correct group-assignment (p<0.0001) based on the levels of expression (GFMI and MFI) of CD57 and the various NKRs. In this case, major contributors of separation between the two groups were NKG2A-GMFI and NKG2D-MFI on CD8 T cells, CD57-GMFI on CD4 T cells, and GMFI/MFI-CD56 on DN T cells. A minor difference between these T cell phenotype-based models was the broader range of distribution of discrimination scores in the cell subset-based model, compared to the narrower score distribution in the receptor expression-based model.

***Figure 2E*** shows a simple linear model of group-discrimination with 66.7% correct group-assignment (p<0.02) based on humoral factors. A stepwise model with 62.7% correct group-assignment was indicated, but such model did not reach statistical significance. This was in marked contrast to data in ***Figure 2B*** indicating contribution of humoral factors in a stepwise model for separation of the impaired and unimpaired groups based on composite cellular and humoral parameters, suggesting interplay between humoral and cellular factors.

### Distinctive immunologic profiles of impaired and unimpaired CHS All Stars elders

The notion for the likely interaction(s) of humoral and cellular factors is line with the well recognized pleiotropic effects of cytokines and chemokines on various cell types including lymphocytes [41]. Hence, factor analysis was performed to determine clusters of T cell and humoral parameters that distinguished impaired from unimpaired elders. Of the 112 immune parameters measured (*Tables S1, S2, S3, S4*), six component factors emerged as distinguishing features for each group. ***Table 2*** shows that these top six factors accounted for >75% of the immune profile (indicated as %CEV) of each group. In both groups, the cellular components of their respective immune profiles were particular NKRs expressed on various T cell subsets, i.e. CD4, CD8, and DN. Such T cell subsets were generally CD28^null^ and/or CD57^+^ consistent with chronologic aging (as shown in ***Figure 1***). Whereas cellular factors accounted for at least 70% of the immune profile of each group, humoral factors accounted for 5.5% of the group-profiles.

**Table 2:** T cell and cytokine profiles of unimpaired and impaired elders.

Unimpaired			Impaired		
Ranked factor #^a^	T cell^b^ or humoral parameter	CEV (%)^c^	Ranked factor #^a^	T cell^b^ or humoral parameter	CEV (%)^c^
1	CD56^+^CD28^null^ cell frequency, and CD56-MFI/GMFI in all subsets	15.3	1	CD158a^+^ cell frequency, and CD158e-MFI/GMFI in all subsets	26.3
2	NKG2D^+^ cell frequency, and NKG2D-MFI/GMFI in all subsets	54.4	2	CD28^null^ cell frequency in CD8 T cells only	45.2
3	CD56^+^CD57^+^CD28^null^ cell frequency, CD56-MFI/GMFI, and CD57-MFI/GMFI in CD4 T cells only	58.7	3	NKG2A^+^ cell frequency, and NKG2A-GMFI in all subsets	58.5
4	CD16^+^ cell frequency, and CD16-MFI/GMFI in all subsets	63.1	4	Co-existing Th1 (IFN-γ, TNF-α) and Th2 (IL-6)	64.0
5	CD56^+^CD57^+^CD28^null^ cell frequency, CD56-MFI/GMFI, and CD57-MFI/GMFI in CD8 T cells only	69.8	5	CD158e^+^ cell frequency, and CD158e-MFI/GMFI in CD8 T cells only	73.2
6	Co-existing Th1 (IL-12p70), and Th2 (IL-5, IL-13)	75.3	6	NKG2A^+^ cell frequency, and NKG2A-GMFI in CD8 T cells	76.3

a Listed are the top six factors that emerged from Factor (or Principal Component) Analysis with Varimax rotation and Kaiser normalization. The analysis was performed by parsing T cell and humoral parameters with a level of significance at an Eigen value (EV) = 5.0 or greater. Covariates entered in the analysis were age, gender, and body mass index. From 112 immune parameters (see text) entered into the analysis, those with rotated loading coefficient of at least 0.70 in absolute value were considered as those identifying the factor (principal component).

b T cell subsets, i.e. CD4, CD8, and DN, were defined by the frequency cells lacking CD28 and/or expressing CD16, CD56, CD57, CD158a, CD158e, NKG2A, and NKG2D, and as well as by the actual level of expression of each of the latter seven antigens (expressed as MFI and GFMI; see Materials and Methods).

c Cumulative Eigen value; 75% CEV was used as a quantitative cut off to define the composite T cell-humoral profile of the groups.

Nevertheless, the two groups had distinct immune profiles (***Table 2***). The cellular profile of the impaired group was defined by CD4, CD8, and DN T cell subsets that expressed inhibitory NKRs, namely; CD158a, CD158e and NKG2A. CD158a^+^ and NKG2A^+^ T cells (Factors #1, # 3) comprised >39% of the impaired group-profile. And CD8 T cell subsets that expressed either CD158e or NKG2A (Factors #5, #6) independently contributed additional 9.2% and 3.2%, respectively, to the group profile. In contrast, the unimpaired group had a cellular profile defined by T cell subsets expressing stimulatory NKRs, namely; CD16, CD56, and NKG2D. These T cell subsets (Factors #1, #2, #4) comprised >58% of the unimpaired group-profile. And CD56-bearing CD4 and CD8 T cells (Factors #3, #5) independently contributed additional 4.3% and 6.7%, respectively, to the group-profile.

As to humoral components, the cytokines IL-6, IFN-γ, and TNF-α were major constituents of the impaired group-profile. In contrast, the cytokines IL-12p70, IL-5, and IL-13 constituted the unimpaired group-profile. Ranking order of the cytokine component of the immune profiles were 4^th^ and 6^th^ for the impaired and unimpaired groups, respectively.

### T cell and cytokine predictors of unimpaired phenotype

To further ascertain relevance of cellular and humoral factors in defining immune fingerprints of the impaired and unimpaired groups, logistic regression analysis was performed. In this case, the analysis was conducted identify immune predictors of unimpaired phenotype. ***Table 3*** shows a stepwise backward logistic model where seven T cell subsets and two cytokines were identified as significant predictors of unimpaired phenotype.

**Table 3:** T cell and humoral predictors of “Unimpaired” phenotype.

	β^a^	Odds Ratio^a^	P^a^
	(standard error of β)	(95% confidence interval)	
T cell subsets^b^			
CD28^null^ (CD4 only)	0.066 (0.320)	1.068 (1.003–1.138)	0.040
CD56^+^CD57^+^ (CD4 only)	0.871 (0.389)	0.419 (0.195–0.898)	0.025
CD56^+^CD57^+^CD28^null^ (CD4 only)	1.254 (0.523)	3.503 (1.256–9.769)	0.017
CD158a^+^ (CD4 only)	−2.030 (1.070)	0.131 (0.016–1.070)	0.045
CD16^+^ (CD4 only)	0.291 (0.088)	1.338 (1.125–1.591)	0.001
CD28^null^ (CD8 only)	−0.189 (0.060)	0.828 (0.737–0.931)	0.002
NKG2A^+^ (CD8 only)	−0.057 (0.019)	0.945 (0.911–0.980)	0.002
Humoral parameters			
IL-5	1.588 (0.652)	4.894 (1.363–17.57)	0.015
IFN-γ	−0.02 (0.008)	0.982 (0.967–0.997)	0.021

a The indicated β-value, odds ratios, and P-values were calculated by logistic regression analysis (in step-wise backward models). Out of 112 immune parameters (see text) entered in the analysis, data shown were 9 T cell and humoral parameters that were significant in the last regression step. Covariates entered in the analysis were age, gender, and body mass index.

b T cell parameters refer to combined measurement of cell frequency, and corresponding GMFI/MFI values for CD16, CD56, CD57, CD158a, and NKG2A for the indicated CD4 or CD8 subset.

Of the T cell subsets, four were positive predictors as indicated by +β values. Of these predictors, CD4^+^CD56^+^CD57^+^CD28^null^ and CD4^+^CD16^+^ T cell subsets had the highest odds ratios at 3.5 and 1.3, respectively. There were also three additional T cell subsets that were negative predictors with −β values, namely CD8^+^NKG2A^+^ T cells, CD8^+^CD28^null^ T cells, and CD4^+^CD158a^+^ T cells. The latter had the lowest odds ratio of 0.131. These data suggested that for each 1.0% increase in the frequency of CD4 T cells expressing CD16 or CD56, or each 1.0 MFI/GMFI unit increase in CD56/CD16 expression, an elder carrying such CD4 T cells would be 1.3 to 3.5 times (or 130% to 350%) more likely be physically/cognitively unimpaired. And for each 1.0% increased prevalence of CD4^+^CD158a^+^ T cells, or each 1.0 MFI/GMFI unit increase in CD158a expression, an elder with such CD4 T cells would be 0.131 times less likely be physically/cognitively unimpaired; or equivalently, 7.63 times (i.e. 1/0.131 = 7.63 or 763%) more likely to be impaired.

Among the humoral factors, IL-5 and IFN-γ were the significant positive (+β) and negative (−β) predictors of unimpaired phenotype, respectively. A significantly higher odds ratio at 4.9 was indicated for IL-5 compared to 0.98 for IFN-γ. These data suggested that for each 1.0 pg/ml increase in plasma IL-5, an elder would be 4.9 times (490%) more likely be physically/cognitively unimpaired. And conversely, for each 1 pg/ml increase in plasma IFN-γ, an elder would be 1.02 times (i.e. 1/0.98 = 1.02 or 102%) more likely to be impaired.

### The NKRs CD56 and NKG2D expressed on T cells are effective drivers of cellular activation

**Figure 3:**
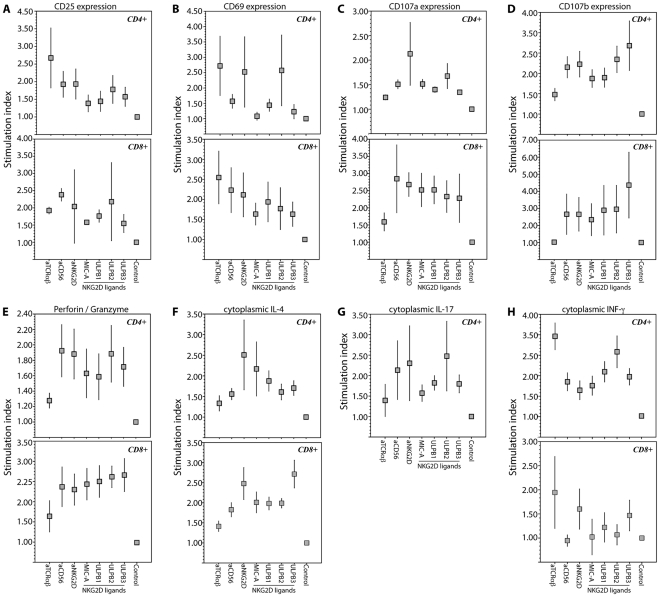
NKRs CD56 and NKG2D expressed on T cells are competent receptors that induce activation of T cells independent of TCR-derived signals. Induction of cell surface expression of activation antigens (**A**) CD25 and (**B**) CD69, markers of granule exocytosis (**C**) CD107a and (**D**) CD107b; and of cytoplasmic stores of (**E**) cytolytic proteins (combined staining for perforin and granzyme), and the cytokines (**F**) IL-4 (**G**) IL-17, and (**H**) IFN-γ in CD4 and CD8 T cells in response to 24-hr incubation in anti-TCRαβ, or anti-CD56, or anti-NKG2D, or recombinant NKG2D ligands MIC-A, ULPB-1, ULPB-2, and ULPB-3. Data shown are means (box) and standard deviations (whiskers) from 13-17 randomly selected unimpaired subjects. P-values of all of the measured cellular responses were significant (p<0.05, Kruskall-Wallis analysis of variance).

To evaluate biological relevance of the expression of NKRs on T cells, cellular bioassays were conducted. We focused on CD56 and NKG2D as they are among the constituent NKRs of the T cell profiles of CHS All Stars elders shown in ***Tables 2*** ***–*** ***3***. Specifically, we examined the ability of CD56 and NKG2D to elicit T cell effector responses independent of TCR triggering. Results of T cell bioassays are summarized in ***Figure 3*** depicting cellular outcomes of the independent ligation of CD56 or NKG2D.

These bioassays showed five significant observations. First, triggering of CD56 alone or NKG2D alone was sufficient to induce T cell responses without the need of co-ligation of the TCR. Results of independent experiments showed that compared to unstimulated controls, CD56 or NKG2D ligation on T cells resulted in significant increases in cell surface expression of activation antigens CD69 and CD25, and markers of vesicle exocytosis CD107a and CD107b; and for increased cytoplasmic stores of cytolytic granules perforin/granzyme, and cytokines IL-4 and IFN-γ.

Second, the magnitudes of TCR-driven T cell responses were highly variable consistent with well documented inefficiency of TCR signaling with chronologic aging [20]. TCR ligation elicited higher levels of responses only in the cases for the expression of IFN-γ, CD25, and CD69 in both CD4 and CD8 T cells. In contrast, CD56 or NKG2D ligation more vigorously induced expression of cytolytic granules, vesicle exocytosis markers, and IL-4.

Third, cytolytic activity indicated by expression of granzyme/perforin, CD107a, and CD107b, whether induced by TCR ligation or by CD56/NKG2D ligation, was observed in both CD4 and CD8 T cells. This was an observation consistent with previous reports that chronologic aging is also associated with novel killing activity of aged CD4 T cells [36], in contrast to conventional CD4 and CD8 T cells of the young that are considered as helper and cytolytic cells, respectively.

Fourth, the magnitudes of T cell responses elicited by triggering of CD56 or NKG2D were not always equivalent. The data showed that NKG2D ligation induced higher levels of cytoplasmic stores of IL-4 in both CD4 and CD8 T cells, and IFN-γ in CD8 T cells; and also higher levels of cell surface CD69 in CD4 T cells. However, activation of the cytolytic machinery indicated by CD107a, CD107b, and perforin/granzyme was equivalently induced by the independent ligation of CD56 and NKG2D.

Fifth, NKG2D-mediated responses induced by its ligands MIC-A and the three ULPBs were also not always equivalent. ULPB3-NKG2D interaction induced higher levels of CD107b in both CD4 and CD8 T cells, and higher IL-4 stores in CD8 T cells. ULBP2-NKG2D interaction induced higher levels of CD69 and perforin/granzyme in CD4 T cells. And MIC-A-NKG2D interaction induced higher stores of IL-4 in CD4 T cells.

## Discussion

### Defining exceptional aging by systems approach: Integration of physical, cognitive, and immunologic parameters

Measurements of physical and cognitive performance have been instrumental in refining the concept of exceptional or successful aging [2], [42]. A basic definition of exceptional aging has been proposed as survival beyond the median lifespan with favorable health and performance despite a long history of diseases and/or concurrent subclinical conditions [2]. Since organ-systems develop and mature at different rates across the lifespan [7], [8], exceptional aging is likely to be mediated by remodeling of physiologic systems that compensate for the normal loss of cell/tissue structure/function with age. Indeed, normal chronologic aging invariably results in structural brain damage including the overall contraction of white and gray matter volumes. But despite such overt damage, some elders can still retain executive brain function due to compensatory higher functional activity of the right posterior parietal cortex [43]. Similarly, advancing age normally results in muscle fiber atrophy and in reduced numbers of motor units that contribute to physical disability. But some elders can still retain physical function with optimal motor endplate conduction due to compensatory increases in the numbers of muscle fibers per motor unit, of pre-synaptic nerve terminal branches, and of post-synaptic neurotransmitter receptors [44]. Thus, there is not only age-related beneficial remodeling in brain and in muscle, but that remodeling appears coordinated as evidenced by the tight association of physical and cognitive ability in prognosticating long-term functional independence and mortality among old people [45], [46].

We have postulated that the immune repertoire may also undergo remodeling with age [17]. As immunity is a determinant of individual fitness, of interest is whether continued physical and cognitive function among elders is linked to an immunological profile distinct from those with poorer performance. Hence, we examined CHS All Stars elders who represent a group of highly functioning, community-dwelling elders with a late life trajectory towards either continued good health or slow functional decline [21]. Such life trajectory of this group of elders is based on results on continuous clinical monitoring spanning nearly two decades [42].

In this study, we defined two categories of CHS All Stars elders based on ADL and 3MSE performance scores. ADL and 3MSE are global measures of physical and cognitive functions, respectively, that have proven to be useful in distinguishing groups of elderly people with either intact function or those with mild disability in large cohort studies [30], [31]. Despite the relatively small number of CHS All Stars subjects (n = 140) examined in this study, along with their wide variations of medical history (***Table 1***), our data show that physically and cognitively unimpaired or impaired elders are identifiable. Confidence for this 3MSE/ADL-based categorization of CHS All Stars elders is provided by the observed group-differences in both gait speed and grip strength. The latter two physical parameters have been validated as independent predictors of long-term function and eventual mortality in the elderly [27], [47].

Consistent with their mean age of 86 years, CHS All Stars elders display the predicted biological indicators of aging in the immune system (***Figure 1***). Their T cell repertoire consists of large populations of CD4 and CD8 T cells lacking expression of CD28 with corresponding gain of expression of CD57 [36], [37]. Such T cells variably express p16, p53, γH2AX and pRB, proteins expressed by somatic cells that are in advanced stages of senescence [48], [49], [50]. CHS All Stars elders also have a global, low-level upregulation of several plasma cytokines and chemokines (*Table S1*) in addition to IL-1, IL-6, TNF-α, and CRP. Low level elevations of the latter four molecules have been the basis for the idea that chronologic aging promotes elaboration of an adverse inflammatory milieu [51], with IL-6 as the most consistent predictor of disability in many elderly populations [32]. But considering the many possible sources of these humoral factors, and their pleiotropic effects including immune-enhancing and pathological properties, we reasoned that immune profiles of highly functioning CHS All Stars elders would more likely consist of a combination of humoral and cellular factors rather than a single factor.

Our data show that T cell and humoral parameters are key variables in parsing the two groups of CHS All Stars elders. Such parameters account for 60% to 80% of the separation between the unimpaired and impaired groups (***Figures 2B–2E***). And combining the same parameters with physical performance measures of gait speed and grip strength account for more than 90% of the separation between the two groups (***Figure 2A***). Moreover, we found that 6 composite T cell and humoral factors, out of the 112 immunological parameters measured (*Tables S1, S2, S3, S4*), differentiate unimpaired elders from those who are physically/cognitively impaired. Our confidence on the identification of these T cell and humoral components of the group-immune profiles came from two independent analytic procedures, namely; factor analysis (***Table 2***) and logistic regression modeling (***Table 3***).

### Global systemic cytokine upregulation in old age deviating from the Th1-Th2-Th17 paradigm

In line with previous studies [32], our data show that the impaired group is associated with the dominance by TNF-α and IL-6 (***Table 2***). IFN-γ is also part of this impaired group profile, suggesting a cytokine environment in the impaired group is Th1 dominant. This is an unexpected finding since IFN-γ is an immune-enhancing cytokine that promotes normal anti-viral activities of monocytes, tissue macrophages, and cytotoxic T cells [52]. However, IFN-γ is also a potent inhibitor of cell proliferation [53], a property that is perhaps consistent with its inclusion in the impaired group-immune profile.

In contrast, the unimpaired group-humoral profile is associated with two Th2 cytokines IL-5 and IL-13 (***Table 2***), although IL-5 is a stronger positive predictor of the unimpaired phenotype (***Table 3***). The group-profile includes co-expression of IL-4 (*Table S1*). Considering that IL-4 is a regulator of IL-5 and IL-13 production that in turn positively feedback IL-4 production [54], these data indicate a likely dominant Th2 cytokine environment in the unimpaired group. This suggestion is supported by the fact that IFN-γ, a Th1 cytokine, is a negative predictor of unimpaired phenotype (***Table 3***). We also found that IL-12p70, a known upstream regulator of IFN-γ, is part of this unimpaired humoral profile (***Table 2***).

Nevertheless, it is important to note that the seeming Th2 versus Th1 dominance in the unimpaired and impaired group, respectively, are within environments where there are in fact sufficient amounts of Th2 (IL-4, IL-5, IL-13) and Th1 (IFN-γ, IL12-p70) cytokines (*Table S1*). This is in contrast to the Th1/Th2 paradigm where normal immune responses reportedly skew to either one Th-environment, but not the co-occurrence of Th1 and Th2 cytokines, because the two Th pathways counter regulate each other. Thus, it would be of interest to examine whether the physiologic cytokine environment in old age is an exception of this Th1/Th2 paradigm since there is also concurrent IL-17 (Th17) (*Table S1*), although the latter is not significantly associated with either impaired or unimpaired groups. An intriguing notion is whether this prevailing “mixed” Th1, Th2, and Th17 environment is essential to lymphocyte homeostasis so as to prevent lymphopenia since neither the thymus nor the bone marrow of the aged are producing any more new lymphocytes. Perhaps more interestingly, it would be of interest to examine whether concurrent Th1, Th2, and Th17 cytokines, rather than skewing towards one Th-type, is more critical in mounting immune responses in old age. Unraveling how balance of Th cytokines is achieved in old age remains to be examined.

### Anti-viral serology is not a significant contributor of immune profiles of old age

Our data show that all the elders examined have generally high titer antibodies to CMV as well as to VZV and to two EBV antigens (*Table S1*). This is in stark contrast to reports that CMV seropositivity is, by itself, a predictor/indicator of poor health and mortality in some Northern European elderly populations [33]. These differences in experimental results could be related to the rate at which different populations become exposed to CMV. Whereas CMV exposure among Europeans seems to occur more gradually through the lifespan leading to peak CMV seropositivity in late life [55], there is more widespread CMV seroprevalence among Americans due to CMV exposure at an earlier age [56]. A recent study [57] indicates that CMV seropositivity is indeed an insufficient measure of health risk for elderly Americans. However, it may still be of interest to also examine whether the observed lack of association between anti-CMV titers with the two categories of CHS All Stars elders could indicate anti-CMV immunity in this group of elderly Americans. A further study is therefore needed to examine causal relationships between CMV reactivation, CMV viral load, anti-CMV antibody titers, CMV-specific T cells, and functional performance.

### Unique subsets of NK-like T cells in old age

Lending support to the idea about repertoire remodeling in old age [17], our present data show that unimpaired and impaired CHS All Stars elders are distinguished by the preponderance of particular subsets of CD4, CD8, and DN T cells bearing stimulatory and inhibitory NKRs, respectively (***Tables 2*** ***,*** ***3***). As indicated, the DN subset expressed neither CD4 nor CD8, and whether DN cells were derived from cells that lost CD4 or CD8 expression is unknown at this time.

The dominant stimulatory NKRs expressed on T cells include NKG2D, CD16, and CD56. And the dominant inhibitory NKRs expressed on T cells include NKG2A, CD158a, and CD158e. CD16 and CD56 are the prototypic NKRs that are normally used to identify NK cells. Although it remains to be examined how expression of these individual NKRs is regulated in aged T cells, increased expression of NKRs on T cells with advancing age is likely to be genetically programmed. We have reported differences in the fine regulation of particular NKRs between T cells and NK cells [58]. We have reported the results of a cross sectional study documenting the increased expression of CD56 on T cells with chronologic aging [16]. And studies comparing examining “young” and “old” groups, the latter usually defined by the age of 60 years and older, also document varying higher levels of expression of the various NKRs on T cells among older persons [10], [14]. Our present data constitute the first evidence for the association between stimulatory and inhibitory NKRs expressed on T cell subsets with functionally unimpaired and impaired elders.

### Unique pathways of TCR-independent activation of T cells of the aged

For conventional NK cells, stimulatory and inhibitory NKRs signal either activation or suppression of NK effector differentiation, respectively. Considering that advancing age is associated with losses of classical NK and T cell function [11], dominance of stimulatory NKRs on aged T cells could be a way to rescue age-related signaling inefficiency of the classical TCR [20] and help maintain cell-mediated immune responses in old age. Along these lines, our present data show stimulatory NKRs CD56 and NKG2D are indeed signaling-competent receptors for T cell activation (***Figure 3***). We focused on CD56 since the function of this NKR is not yet known, unlike CD16 that is a validated NK cell signaling receptor [59]. We also examined NKG2D since it is a receptor known recognize viral epitopes including CMV [35], albeit NKG2D-CMV interaction was not examined here. Nonetheless, our data show that ligation of either CD56 or NKG2D, independent of TCR ligation, can result in various cellular outcomes including expression of activation antigens (CD25, CD69), cytolytic granules, and cytoplasmic stores of IL-4 and IFN-γ as well as granule exocytosis (indicated by the cell surface expression of CD107a/b). We should note that the cellular bioassays here were aimed at demonstrating the functional competence of CD56 and NKG2D as an independent receptor on T cells. A comparison of the magnitudes of CD56-/NKG2D-driven T cell responses between unimpaired and impaired elders still remains to be examined, an undertaking that may require a much larger cohort.

Remarkably, our data show magnitudes of CD56/NKG2D-driven responses can even be higher than similar responses driven through TCR ligation. Whether the CD56/NKG2D-driven expression of IL-4 and IFN-γ contributes to the observed systemic upregulation of these two cytokines (***Tables 2*** ***,*** ***3***) will have yet to be examined. Similarly, the cellular significance of the expression of inhibitory NKRs NKG2A, CD158a, and CD158e on aged T cells remains to be evaluated as we had limited blood specimens to conduct multiple replicate bioassays for both stimulatory and inhibitory NKRs. However, dominance of inhibitory NKRs on aged T cells could either indicate continued malfunction of the aged T cells, or perhaps an adaptation to control autoreactive T cells in old age [60]. Thus, it remains to be determined whether stimulatory and inhibitory NKR expression on the same T cell counter regulate each other, or that allosteric dominance of one type of NKR would determine the eventual effector function of the NKR-bearing T cell. It also remains to be examined whether the stimulatory NKRs can potentiate TCR-derived signal, which has been documented to be either inefficient or defunct in aged T cells [20].

*In summary*, results of the present cross sectional study demonstrate the value of immunologic parameters in combination with measures of physical and cognitive performance in defining resilience and healthy aging in older adults. Association between T cell subsets expressing stimulatory NKRs, systemic IL-5, and intact functional performance, and conversely, association between inhibitory NKRs, IFN-γ, and mild disability suggest divergent immunopathways. Further elucidation of these pathways will provide better understanding of what constitutes immune competence or incompetence in old age. Longitudinal studies involving follow up of subjects across the lifespan are needed to determine factors that drive elaboration of NKRs on T cells and systemic upregulation of IFN-γ and IL-5. Such studies would also be an opportunity to examine whether and how changes in health and performance might modify expression of these immune-related molecules, or alternatively, to determine whether modulation of expression of these molecules could have direct impact on health in old age.

## Supporting Information

Table S1 **Systemic levels of humoral factors in Impaired and Unimpaired groups of elders.**
(DOC)

Table S2 **Frequency of T cell subsets in Impaired and Unimpaired groups of elders.**
(DOC)

Table S3 **Density of receptor expression measured as mean fluorescence intensity (MFI) on T cell subsets in Impaired and Unimpaired groups of elders.**
(DOC)

Table S4 **Density of receptor expression measured as geometric mean fluorescence intensity (GMFI) on T cell subsets in Impaired and Unimpaired groups of elders.**
(DOC)
