# Supplementary material for: NK-Like T Cells and Plasma Cytokines, but Not Anti-Viral Serology, Define Immune Fingerprints of Resilience and Mild Disability in Exceptional Aging
Source: PLoS One. 2011 Oct 20;6(10):e26558. doi: 10.1371/journal.pone.0026558 (PMC3197651; doi:10.1371/journal.pone.0026558)
Supplement: Table S1 — Systemic levels of humoral factors in Impaired and Unimpaired groups of elders. (DOC) [file pone.0026558.s001.doc]

***Table S1***. Systemic levels of humoral factors in Impaired and Unimpaired groups of elders.

|  | Impaired | | | | | Unimpaired | | | | |
| --- | --- | --- | --- | --- | --- | --- | --- | --- | --- | --- |
|  | Mean | Median | Mode | Range | CV | Mean | Median | Mode | Range | CV |
| *Cytokines* |  |  |  |  |  |  |  |  |  |  |
| IL-1β (pg/ml) | 0.98 | 0.31 | 0.25 | 21.43 | 2.89 | 1.01 | 0.4 | 0.28 | 21.21 | 2.41 |
| IL-2 (pg/ml) | 5.0 | 0.0 | 0.0 | 94.0 | 3.55 | 1.0 | 0.0 | 0.0 | 53.0 | 5.07 |
| IL-4 (pg/ml) | 1.28 | 0.21 | 0.0 | 33.55 | 3.68 | 0.36 | 0.2 | 0.0 | 2.93 | 1.35 |
| IL-5 (pg/ml) * | 0.42 | 0.23 | 0.0 | 3.31 | 1.31 | 1.49 | 1.4 | 0.0 | 4.08 | 0.11 |
| IL-6 (pg/ml) *** | 13.94 | 1.85 | 0.0 | 485.17 | 0.60 | 3.6 | 2.46 | 0.0 | 26.68 | 1.31 |
| IL-7 (pg/ml) * | 18.44 | 7.57 | 0.0 | 539.94 | 3.84 | 6.44 | 5.77 | 3.15 | 23.07 | 0.71 |
| IL-10 (pg/ml) * | 5.69 | 0.96 | 0.95 | 226.08 | 5.23 | 1.17 | 0.94 | 0.72 | 6.41 | 0.79 |
| IL-12 p70 (pg/ml) *** | 46.7 | 2.9 | 0.0 | 1436.7 | 0.56 | 5.1 | 1.4 | 0.0 | 31.7 | 1.64 |
| IL-13 (pg/ml) * | 0.87 | 0.44 | 0.0 | 14.57 | 2.24 | 0.65 | 4.4 | 0.18 | 3.54 | 1.09 |
| IL-17 (pg/ml) | 7.0 | 0.0 | 0.0 | 128.0 | 2.74 | 8.0 | 0.0 | 0.0 | 44.0 | 3.25 |
| IFN-γ (pg/ml) *** | 183.41 | 18.03 | 4.92 | 6845.33 | 0.65 | 23.59 | 11.85 | 0.0 | 174.62 | 1.37 |
| TNF-α (pg/ml) * | 11.0 | 0.0 | 0.0 | 409.0 | 4.78 | 5.0 | 0.0 | 0.0 | 68.0 | 2.35 |
| *Chemokines* |  |  |  |  |  |  |  |  |  |  |
| IL-8 (pg/ml) | 3.48 | 2.57 | 0.0 | 18.49 | 1.14 | 4.75 | 2.28 | 0.0 | 87.17 | 2.21 |
| GCSF (pg/ml) *** | 113.8 | 19.85 | 0.0 | 1899.1 | 0.37 | 29.7 | 16.77 | 8.44 | 341.13 | 1.58 |
| GMCSF (pg/ml) | 2.0 | 0.0 | 0.0 | 48.0 | 4.06 | 1.0 | 0.0 | 0.0 | 30.0 | 4.56 |
| MCP-1 (pg/ml) | 13.7 | 11.0 | 8.0 | 41.10 | 0.74 | 13.6 | 10.0 | 7.8 | 93.2 | 0.99 |
| MIP-1β (pg/ml) * | 49.5 | 32.6 | 4.0 | 916.70 | 2.37 | 38.0 | 31.8 | 31.5 | 140.0 | 0.68 |
| *Acute phase reactant* |  |  |  |  |  |  |  |  |  |  |
| CRP (pg/ml) | 7.0 | 2.0 | 1.0 | 162.0 | 3.08 | 5.0 | 2.0 | 1.0 | 62.0 | 1.82 |
| *Anti-viral antibody* |  |  |  |  |  |  |  |  |  |  |
| EBV-EA IgG (RU/ml) | 14.32 | 1.84 | 1.07 | 617.1 | 0.86 | 2.53 | 2.01 | 1.55 | 7.5 | 0.73 |
| EBV- EBNA IgG (RU/ml) | 249.08 | 195.05 | 824.0 | 824.0 | 5.64 | 244.03 | 213.69 | 0.0 | 824.0 | 0.64 |
| CMV IgG index | 1.6 | 1.72 | 1.72 | 2.36 | 0.34 | 1.65 | 1.73 | 1.63 | 2.1 | 0.25 |
| CMV IgG (IU/ml) | 41.42 | 12.02 | 0.10 | 277.93 | 1.39 | 37.29 | 13.48 | 0.11 | 188.12 | 1.31 |
| VZV IgG (IU/ml) | 1.19 | 0.97 | 0.57 | 4.59 | 0.71 | 1.07 | 0.85 | 0.38 | 5.05 | 0.76 |
| Flu HA IgG index | 6.0 | 2.0 | 1.0 | 56.0 | 1.82 | 11.0 | 3.0 | 2.0 | 97.0 | 2.17 |

*Mean or median values that are different (but not statistically significant) between the two groups.

***Statistically different mean values at P<0.05 (two-tailed *t*-test with adjustment for pairwise comparisons using Bonferroni correction).
