# Supplementary material for: NK-Like T Cells and Plasma Cytokines, but Not Anti-Viral Serology, Define Immune Fingerprints of Resilience and Mild Disability in Exceptional Aging
Source: PLoS One. 2011 Oct 20;6(10):e26558. doi: 10.1371/journal.pone.0026558 (PMC3197651; doi:10.1371/journal.pone.0026558)
Supplement: Table S2 — Frequency of T cell subsets in Impaired and Unimpaired groups of elders. (DOC) [file pone.0026558.s002.doc]

***Table S2***. Frequency of T cell subsets in Impaired and Unimpaired groups of elders.

|  | Impaired | | | | | Unimpaired | | | | |
| --- | --- | --- | --- | --- | --- | --- | --- | --- | --- | --- |
|  | Mean | Median | Mode | Range | CV | Mean | Median | Mode | Range | CV |
| Ratio CD4^+^ to CD8^+^ T cells | 3.2 | 2.47 | 3.5 | 9.63 | 0.71 | 2.91 | 2.29 | 0.76 | 9.64 | 0.64 |
| % CD4^+^ T cells | 43 | 42 | 56 | 49 | 0.24 | 43 | 40 | 32 | 55 | 0.27 |
| % CD8^+^ T cells | 18 | 17 | 19 | 40 | 0.47 | 19 | 17 | 11 | 34 | 0.43 |
| % DN T cells | 39 | 39 | 24 | 52 | 0.30 | 38 | 37 | 34 | 51 | 0.28 |
| *Frequency of CD4^+^ T cell subsets* | | | | | | | | | | |
| CD28^null^ *** | 59 | 60 | 70 | 60 | 0.25 | 65 | 64 | 64 | 61 | 0.21 |
| CD57^+^ *** | 9 | 7 | 1 | 27 | 0.66 | 12 | 9 | 5 | 46 | 0.10 |
| CD28^null^CD57^+^ *** | 5 | 4 | 3 | 18 | 0.72 | 9 | 6 | 14 | 41 | 0.12 |
| CD56^+^ | 26 | 24 | 20 | 47 | 0.42 | 23 | 21 | 16 | 46 | 0.36 |
| CD56^+^CD57^+^ | 5 | 4 | 0 | 15 | 0.70 | 7 | 5 | 1 | 40 | 1.05 |
| CD28^null^CD56^+^CD57^+^ *** | 3 | 3 | 1 | 12 | 0.75 | 25 | 24 | 22 | 34 | 0.13 |
| NKG2D^+^  * | 17 | 15 | 14 | 58 | 0.54 | 25 | 24 | 11 | 36 | 0.46 |
| NKG2A^+^ | 19 | 14 | 13 | 97 | 0.99 | 15 | 14 | 13 | 33 | 0.50 |
| CD16^+^ | 4 | 3 | 1 | 33 | 1.18 | 4 | 4 | 1 | 29 | 0.95 |
| CD158a^+^ | 1 | 0 | 0 | 49 | 4.60 | 0 | 0 | 0 | 3 | 0.94 |
| CD158b^+^ | 2 | 1 | 0 | 20 | 2.00 | 3 | 1 | 0 | 35 | 2.39 |
| CD158e^+^ | 2 | 1 | 0 | 22 | 1.55 | 2 | 1 | 0 | 53 | 2.59 |
| *Frequency of CD8^+^ T cell subsets* | | | | | | | | | | |
| CD28^null^ | 75 | 75 | 75 | 37 | 0.11 | 77 | 78 | 80 | 40 | 0.11 |
| CD57^+^ *** | 47 | 46 | 61 | 64 | 0.30 | 54 | 53 | 34 | 70 | 0.28 |
| CD28^null^CD57^+^ *** | 37 | 36 | 36 | 45 | 0.30 | 42 | 42 | 44 | 55 | 0.29 |
| CD56^+^ | 43 | 39 | 27 | 49 | 0.32 | 40 | 39 | 29 | 61 | 0.32 |
| CD56^+^CD57^+^ | 26 | 23 | 21 | 62 | 0.54 | 28 | 25 | 15 | 63 | 0.44 |
| CD28^null^CD56^+^CD57^+^ * | 20 | 18 | 15 | 44 | 0.52 | 32 | 32 | 32 | 47 | 0.42 |
| NKG2D^+^ * | 15 | 13 | 11 | 34 | 0.54 | 22 | 22 | 22 | 31 | 0.58 |
| NKG2A^+^ *** | 48 | 47 | 47 | 38 | 0.04 | 31 | 31 | 35 | 46 | 0.34 |
| CD16^+^ | 10 | 8 | 1 | 40 | 0.85 | 9 | 7 | 10 | 37 | 0.81 |
| CD158a^+^ * | 12 | 11 | 10 | 21 | 1.86 | 5 | 5 | 5 | 35 | 3.13 |
| CD158b^+^ | 9 | 6 | 12 | 47 | 0.94 | 9 | 6 | 2 | 38 | 0.88 |
| CD158e^+^ * | 15 | 13 | 13 | 28 | 1.18 | 4 | 3 | 2 | 38 | 1.26 |
| *Frequency of DN T cell subsets* | | | | | | | | | | |
| CD28^null^ | 84 | 86 | 86 | 35 | 0.09 | 86 | 87 | 79 | 31 | 0.07 |
| CD57^+^ | 22 | 20 | 17 | 53 | 0.54 | 24 | 23 | 22 | 45 | 0.45 |
| CD28^null^CD57^+^ | 18 | 17 | 17 | 38 | 0.54 | 20 | 19 | 14 | 39 | 0.46 |
| CD56^+^ *** | 44 | 42 | 38 | 50 | 0.28 | 39 | 38 | 36 | 57 | 0.29 |
| CD56^+^CD57^+^ | 17 | 15 | 12 | 52 | 0.65 | 17 | 15 | 12 | 40 | 0.53 |
| CD28^null^CD56^+^CD57^+^ | 14 | 12 | 11 | 37 | 0.64 | 15 | 13 | 11 | 33 | 0.52 |
| NKG2D^+^ | 15 | 14 | 16 | 45 | 0.48 | 14 | 13 | 11 | 31 | 0.51 |
| NKG2A^+^ *** | 23 | 20 | 11 | 80 | 0.10 | 18 | 16 | 15 | 45 | 0.45 |
| CD16^+^ | 24 | 21 | 13 | 67 | 0.57 | 6 | 4 | 4 | 55 | 0.51 |
| CD158a^+^ | 2 | 1 | 1 | 36 | 2.61 | 1 | 1 | 0 | 7 | 1.25 |
| CD158b^+^ | 9 | 7 | 10 | 40 | 0.80 | 10 | 4 | 4 | 55 | 0.89 |
| CD158e^+^ | 7 | 6 | 4 | 42 | 0.97 | 23 | 21 | 11 | 53 | 1.15 |

* Mean or median values are different (but not statistically significant) between the two groups.

***Statistically different mean values at P<0.05 (two-tailed *t*-test with adjustment for pairwise comparisons using Bonferroni correction).
