# Supplementary material for: NK-Like T Cells and Plasma Cytokines, but Not Anti-Viral Serology, Define Immune Fingerprints of Resilience and Mild Disability in Exceptional Aging
Source: PLoS One. 2011 Oct 20;6(10):e26558. doi: 10.1371/journal.pone.0026558 (PMC3197651; doi:10.1371/journal.pone.0026558)
Supplement: Table S3 — Density of receptor expression measured as mean fluorescence intensity (MFI) on T cell subsets in Impaired and Unimpaired groups of elders. (DOC) [file pone.0026558.s003.doc]

***Table S3.*** Density of receptor expression measured as mean fluorescence intensity (MFI) on T cell subsets in Impaired and Unimpaired groups of elders.

|  | Impaired | | | | | Unimpaired | | | | |
| --- | --- | --- | --- | --- | --- | --- | --- | --- | --- | --- |
|  | Mean | Median | Mode | Range | CV | Mean | Median | Mode | Range | CV |
| *MFI expression level on CD4^+^ T cell subsets* | | | | | | | | | | |
| CD56 | 276 | 250 | 147 | 847 | 0.47 | 281 | 237 | 147 | 822 | 0.58 |
| CD57^+^ *** | 4,114 | 2,731 | 533 | 18,253 | 0.99 | 6,175 | 3,047 | 614 | 36,168 | 0.13 |
| NKG2D * | 322 | 194 | 179 | 765 | 0.45 | 325 | 263 | 195 | 3,171 | 0.13 |
| NKG2A | 247 | 168 | 147 | 3022 | 1.70 | 217 | 182 | 136 | 2,631 | 1.33 |
| CD16 * | 828 | 788 | 830 | 1,833 | 0.42 | 936 | 949 | 814 | 1,663 | 0.39 |
| CD158a * | 411 | 378 | 341 | 1,666 | 0.53 | 394 | 384 | 344 | 266 | 0.15 |
| CD158b | 93 | 66 | 63 | 536 | 1.06 | 101 | 67 | 58 | 652 | 1.23 |
| CD158e | 155 | 104 | 117 | 2,481 | 2.06 | 325 | 109 | 102 | 9,845 | 3.59 |
| *MFI expression level on CD8^+^ T cell subsets* | | | | | | | | | | |
| CD56 | 497 | 404 | 281 | 1,186 | 0.52 | 451 | 397 | 223 | 1,079 | 0.42 |
| CD57 | 31,303 | 26,397 | 1,759 | 112,241 | 0.75 | 33,948 | 29,778 | 2,184 | 93,174 | 0.62 |
| NKG2D * | 201 | 282 | 256 | 844 | 0.68 | 349 | 337 | 261 | 484 | 1.00 |
| NKG2A * | 781 | 628 | 249 | 2,342 | 0.05 | 613 | 449 | 189 | 3,436 | 0.27 |
| CD16 | 1,209 | 1,207 | 437 | 3,026 | 0.38 | 1,264 | 1,261 | 1,272 | 2,385 | 0.34 |
| CD158a * | 790 | 740 | 514 | 996 | 0.31 | 736 | 541 | 402 | 13,044 | 1.99 |
| CD158b | 275 | 140 | 117 | 6,188 | 2.95 | 183 | 133 | 119 | 1,071 | 0.79 |
| CD158e * | 239 | 263 | 202 | 836 | 0.13 | 127 | 109 | 76 | 779 | 0.75 |
| *MFI expression on DN T cell subsets* | | | | | | | | | | |
| CD56 *** | 491 | 451 | 39 | 1,050 | 0.44 | 521 | 574 | 323 | 944 | 0.05 |
| CD57 | 10,475 | 9,109 | 811 | 42,975 | 0.79 | 11,389 | 7,723 | 1,090 | 48,302 | 0.83 |
| NKG2D * | 312 | 277 | 235 | 684 | 0.40 | 391 | 379 | 231 | 887 | 0.50 |
| NKG2A * | 275 | 205 | 124 | 2,452 | 1.34 | 222 | 202 | 222 | 1,660 | 0.81 |
| CD16 | 1,887 | 1,849 | 1,336 | 3,531 | 0.30 | 1,845 | 1,809 | 1,508 | 3,482 | 0.31 |
| CD158a | 489 | 447 | 351 | 1,096 | 0.43 | 445 | 431 | 353 | 598 | 0.27 |
| CD158b | 201 | 158 | 113 | 844 | 0.78 | 194 | 144 | 85 | 906 | 0.76 |
| CD158e | 153 | 143 | 4 | 656 | 0.67 | 158 | 127 | 118 | 1,196 | 0.89 |

* Mean or median values that are different (but not statistically significant) between the two groups.

***Statistically different mean values at P<0.05 (two-tailed *t*-test with adjustment for pairwise comparisons using Bonferroni correction).
