# Supplementary material for: NK-Like T Cells and Plasma Cytokines, but Not Anti-Viral Serology, Define Immune Fingerprints of Resilience and Mild Disability in Exceptional Aging
Source: PLoS One. 2011 Oct 20;6(10):e26558. doi: 10.1371/journal.pone.0026558 (PMC3197651; doi:10.1371/journal.pone.0026558)
Supplement: Table S4 — Density of receptor expression measured as geometric mean fluorescence intensity (GMFI) on T cell subsets in Impaired and Unimpaired groups of elders. (DOC) [file pone.0026558.s004.doc]

***Table S4***. Density of receptor expression measured as geometric mean fluorescence intensity (GMFI) on T cell subsets in Impaired and Unimpaired groups of elders.

|  | Impaired | | | | | Unimpaired | | | | |
| --- | --- | --- | --- | --- | --- | --- | --- | --- | --- | --- |
|  | Mean | Median | Mode | Range | CV | Mean | Median | Mode | Range | CV |
| *GMFI expression level on CD4^+^ T cell subsets* | | | | | | | | | | |
| CD56 | 168 | 149 | 133 | 587 | 0.51 | 179 | 144 | 143 | 789 | 0.62 |
| CD57^+^ *** | 502 | 467 | 290 | 659 | 0.28 | 948 | 476 | 345 | 4,930 | 0.11 |
| NKG2D | 158 | 138 | 115 | 353 | 0.44 | 187 | 136 | 123 | 3,204 | 1.89 |
| NKG2A | 176 | 113 | 144 | 2,507 | 1.98 | 172 | 126 | 115 | 2,555 | 1.65 |
| CD16 *** | 523 | 487 | 224 | 1,285 | 0.51 | 622 | 608 | 382 | 1,235 | 0.05 |
| CD158a | 338 | 311 | 275 | 1,176 | 0.46 | 332 | 326 | 301 | 243 | 0.16 |
| CD158b | 64.9 | 50.7 | 45.3 | 490.3 | 1.22 | 69.7 | 49.9 | 49.4 | 589.4 | 1.29 |
| CD158e | 56 | 56 | 46 | 194 | 0.62 | 68 | 59 | 49 | 495 | 0.86 |
| *GMFI expression level on CD8^+^ T cell subsets* | | | | | | | | | | |
| CD56 | 258 | 222 | 144 | 589 | 0.49 | 247 | 226 | 136 | 615 | 0.43 |
| CD57 *** | 5,741 | 2,911 | 1,663 | 28,082 | 1.11 | 8,731 | 4,425 | 534 | 37,386 | 0.11 |
| NKG2D | 130 | 114 | 97 | 310 | 0.53 | 101 | 90 | 144 | 275 | 0.55 |
| NKG2A *** | 311 | 293 | 228 | 673 | 0.05 | 281 | 273 | 226 | 424 | 0.29 |
| CD16 *** | 784 | 769 | 881 | 1,839 | 0.43 | 877 | 863 | 684 | 1,552 | 0.04 |
| CD158a | 429 | 406 | 388 | 802 | 0.04 | 430 | 412 | 347 | 856 | 0.28 |
| CD158b | 96 | 80 | 65 | 764 | 1.03 | 92 | 79 | 64 | 234 | 0.43 |
| CD158e | 66 | 62 | 76 | 201 | 0.08 | 68 | 61 | 32 | 307 | 0.62 |
| *GMFI expression level on DN T cell subsets* | | | | | | | | | | |
| CD56 | 259 | 232 | 142 | 524 | 0.42 | 236 | 293 | 179 | 479 | 0.41 |
| CD57 | 1,013 | 719 | 267 | 5,641 | 0.88 | 1,064 | 806 | 518 | 4,865 | 0.78 |
| NKG2D | 138 | 125 | 107 | 266 | 0.41 | 137 | 124 | 124 | 703 | 0.63 |
| NKG2A | 203 | 148 | 133 | 1,968 | 1.39 | 179 | 156 | 139 | 1,534 | 0.94 |
| CD16 | 1,300 | 1,239 | 1,129 | 2,556 | 0.32 | 1,308 | 1,281 | 930 | 2,220 | 0.30 |
| CD158a | 339 | 325 | 287 | 964 | 0.43 | 312 | 306 | 315 | 380 | 0.23 |
| CD158b | 88 | 78 | 76 | 193 | 0.41 | 89 | 80 | 62 | 205 | 0.47 |
| CD158e | 73 | 71 | 102 | 240 | 0.59 | 78 | 73 | 66 | 473 | 0.76 |

* Mean or median values that are different (but not statistically significant) between the two groups.

*** Statistically different mean values at P<0.05 (two-tailed *t*-test with adjustment for pairwise comparisons using Bonferroni correction).
